# In Silico Analysis of USP7 Inhibitors Based on Building QSAR Models and Fragment Design for Screening Marine Compound Libraries

**DOI:** 10.3390/md22010001

**Published:** 2023-12-19

**Authors:** Huiting Tan, Chenying Li, Tianli Lai, Lianxiang Luo

**Affiliations:** 1 The First Clinical College, Guangdong Medical University, Zhanjiang 524023, China; tanhui@gdmu.edu.cn (H.T.); ayr66@gdmu.edu.cn (C.L.); laitianli@gdmu.edu.cn (T.L.); 2 The Marine Biomedical Research Institute, Guangdong Medical University, Zhanjiang 524023, China; 3 The Marine Biomedical Research Institute of Guangdong Zhanjiang, Zhanjiang 524023, China

**Keywords:** marine natural compounds, USP7, QSAR, molecular docking, scaffold hopping, virtual screening, molecular dynamics

## Abstract

USP7 is highly expressed in a variety of tumors and is thought to play a major role in cancer development. However, there are no drugs available to target USP7, so there is a need to develop new USP7 inhibitors. In this study, AutoQSAR, multiple linear regression, and Naive Bayesian models were constructed using 543 compounds and used to analyze marine compounds. After selecting 240 small molecules for molecular docking with Maestro, MOE, and GOLD, better small molecules than the positive compound P217564 were screened. The molecular structure of “1, 2-dibromobenzene” was optimized to improve the binding effect of the protein, and 10 optimized compounds in ADMET performed well during the screening process. To study the dynamic combination of protein–ligand effect consistency with static molecular docking, 100ns molecular dynamics simulations of candidate compound 1008-1, reference compound P217564, and negative-positive GNE2917 were conducted. The results of molecular docking and molecular dynamics simulation analysis showed that compound 1008-1 maintained a stable conformation with the target protein. Thus, the comprehensive analysis suggests that compound 1008-1 could provide new possibilities for USP7 covalent inhibitor candidates.

## 1. Introduction

Ubiquitination is a significant mechanism for managing the stability of the majority of proteins in the cell, and it is also essential for the cell cycle and other cellular processes, DNA repair, and cell death. It is also an important pathway for protein post-translational modification [1,2,3,4]. Deubiquitinating enzymes (DUBs) can block the ubiquitination pathway, as they remove ubiquitin from proteins and, if inhibited, can lead to the degradation of specific proteins [5]. According to the structure of the catalytic domain of deubiquitination enzymes, deubiquitination enzymes can be divided into the following seven subfamilies: Ubiquitin-specific proteases (USPs), ubiquitin carboxyl-terminal hydrolases (UCHs), ovarian tumor proteases (OTUs), Josephins, the JAB1/MPN/MOV34 family, Zinc finger UB-specific proteases (ZUP/ZUFSP), and monocyte chemotactic protein-inducing proteins (MCPIP) [6,7].

Ubiquitin-specific proteases (USPs) are the largest family of ubiquitinating enzymes [8]. Ubiquitin-specific protease 7 (USP7), also known as herpes virus-associated protease (HAUSP), has received widespread attention in recent years due to its close association with the occurrence and development of a variety of cancers, including breast cancer, ovarian cancer, prostate cancer, cervical cancer, and colorectal cancer [9,10,11,12]. USP7 was the first USP found to bind and stabilize p53 and is one of the most widely studied. USP7 can increase the level of MDM2, the negative regulatory protein of the tumor suppressor gene p53, and then decrease the level of p53 [13]. USP7 has multiple roles in regulating the p53 pathway, especially in p53-dependent cellular stress responses [14,15,16]. Inhibition of USP7 leads to degradation of the E3 ligase MDM2, which in turn reactivates the tumor suppressor p53 in various cancers [5]. In addition, USP7 is involved in the regulation of several key signal transduction proteins associated with cancer development, such as PTEN, FoxO4, HIF-1α, and PHF8 [9,10,17,18,19,20]. Taken together, USP7 is a promising target for anticancer therapy. Over the past decade, many researchers have made great efforts to search for USP7 inhibitors, but no related inhibitors have entered clinical trials. According to the different modes of action, USP7 inhibitors are mainly divided into three categories: (a) inhibitors that do not directly bind to inhibit USP7 activity, such as P5091, which accelerate their proteasome degradation by promoting ubiquitination of MDM2 and MDMX [21]; (b) covalent binding inhibitors, such as P22077, covalently modify cysteine 223 in the catalytic center of USP7, causing conformational changes in the active site and inhibiting its enzyme activity [22]; (c) allosteric inhibitors, such as XL188, can bind directly to the ubiquitin-binding site of USP7 or are domain-specific to inhibit the activity of its deubiquitinating enzyme [23]. Some of the currently developed USP7 inhibitors have a narrow kinetic window, and although they can achieve the purpose of stabilizing P53, they have obvious side effects and also inhibit unrelated enzymes, showing weak inhibitory activity or containing undesirable chemical properties. Therefore, it is urgent to optimize more efficient and accurate methods to screen new USP7 inhibitors.

In recent years, marine organisms have produced products with unique chemical structures, significant biological activity, and high medicinal value. People continue to explore these marine natural products more deeply [24]. Studies have shown that substances extracted from marine organisms have antitumor, antithrombotic, and antibacterial effects and show surprising activity, which makes it possible to screen out active USP7 inhibitors with novel structures [25,26,27]. In order to make marine compound sources more comprehensive, we have integrated three databases related to marine natural products: (a) the Marine Natural Products Database (MNPD); (b) the Comprehensive Marine Natural Products Database (CMNPD); and (c) the Seaweed Metabolite Database (SWMD). We hope to use the valuable resources of the ocean to screen for novel USP7 inhibitors.

In the past decades, more and more marketed drugs have added their effects through covalency, and there is growing interest in covalent drugs [28]. The pharmacological advantages of covalent inhibitors are being extensively studied. Covalent inhibitors can obtain longer drug residence times and improve target selectivity compared to non-covalent inhibitors. Drugs that work through covalency, such as Osimertinib, Clopidogrel, and Boceprevir, have been approved by the Food and Drug Administration (FDA) [29]. In the USP7 crystal structure, the catalytic domain is composed of amino acid residues CYS223, HIS464 and ASP481, which are called catalytic triplets and together participate in the substrate deubiquitination process [30]. Some studies have shown that compound FT827 contains a vinyl sulfonamide structure, which can be covalently modified to the amino acid residue CYS223 in the USP7 catalytic domain [5]. Therefore, USP7 structural crystals can provide a structural basis for screening covalent inhibitors and improve selectivity through covalent action.

**Figure 1:**
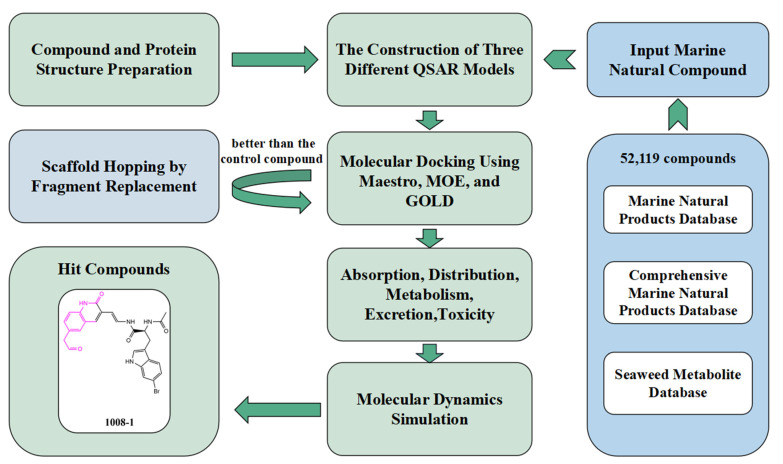
Virtual screening process for USP7 covalent inhibitors: Three marine compound libraries were selected, and the compound library was screened by constructing three different QSAR models to analyze active and inactive compounds. After molecular docking with Maestro, GOLD, and MOE, consensus analysis of Marine compounds was performed, and the hit compounds were optimized by scaffold hopping. Subsequently, another round of molecular docking was performed, and using positive controls, the results before and after docking were analyzed to find out the dominant small molecules. The optimized small molecules were then subjected to pharmacokinetics, and finally, they were each subjected to three molecular dynamics simulations and averaged. Through comprehensive analysis from multiple perspectives, the superior USP7 covalent inhibitor 1008-1 was screened out.

In this study, we sought to identify novel and effective covalent inhibitors of USP7. To this end, we first collected UPS7 inhibitors and evaluated and selected QSAR models using different procedures and different methods: two regression models (the AutoQSAR model and multiple linear regression model), and a Naive Bayesian model. The QSAR model was used to analyze the structural relationship of USP7 inhibitors. Then, the model is used to screen the Marine combinatorial library. Then, the structure-based virtual screening was carried out. Three different docking programs—Maestro; MOE; and GOLD—were used for molecular docking; and the better compounds were screened according to the interactions between the composites. Three different docking programs, Maestro, MOE, and GOLD, were used for molecular docking, and the better compounds were selected according to the interactions between the compounds. We then selected small Marine molecules that were better than the positive control compounds to perform the Scaffold hopping. The compounds before and after structure optimization were compared by molecular docking. The absorption, distribution, metabolism, excretion, and toxicity of the selected compounds were predicted. Finally, molecular dynamics simulations of candidate compound 1008-1, positive and negative compounds, were performed, taking into account the static and dynamic interactions of the crystal complexes, and effective USP7 covalent inhibitors were identified by observing the conformational stability of the complexes. Figure 1 illustrates the workflow of this study.

## 2. Results

### 2.1. The Analysis of Three Different QSAR Models

#### 2.1.1. Construction and Verification of AutoQSAR Model

As more data becomes available over time, updated QSAR models will make the predictive models more accurate. Based on various regression algorithms, 10 AutoQSAR models were constructed by using the small molecules of USP7 inhibitory activity in the public database BindingDB. The 10 models are ranked according to their overall score. As shown in Table 1, these are the statistical parameters of 10 AutoQSAR models. Although the standard deviation and root mean square error of the kpls_linear_20 model and the kpls_dendritic_20 model are lower than other scores, their combined scores are not the best two models. The model with the highest composite score is the model kpls_radial_20, which is the best model produced with a radial fingerprint by the kernel-based partial least squares (KPLS) regression method. The ranking score of kpls_radial_20 is 0.7847, the R-square value (coefficient of determination) of the training set is 0.8077, and the Q-square value of the test set is 0.8016. Compared with other models, the difference between them is the smallest, which is 0.0061, indicating that kpls_radial_20 has stronger generalization ability and can predict unknown small molecules with different activities. Because there are 52,119 compounds in the target compound library to be screened, the AutoQSAR model is required to have stronger generalization ability. Therefore, we chose the kpls_radial_20 model to predict small molecules in the marine compound library.

The automatic quantitative structure-activity relationship model can be applied to the validation set in the same work in which the model is built, or it can be applied to newly acquired compounds at any later point in time. The kpls_radial_20 model was evaluated as the best model for AutoQSAR. Thus, using this model to predict marine compounds, 240 marine small-molecule compounds with pIC50 values greater than 6 were selected for the next step of XP docking and covalent docking.

#### 2.1.2. Construction and Verification of Naive Bayesian Model

**Figure 2:**
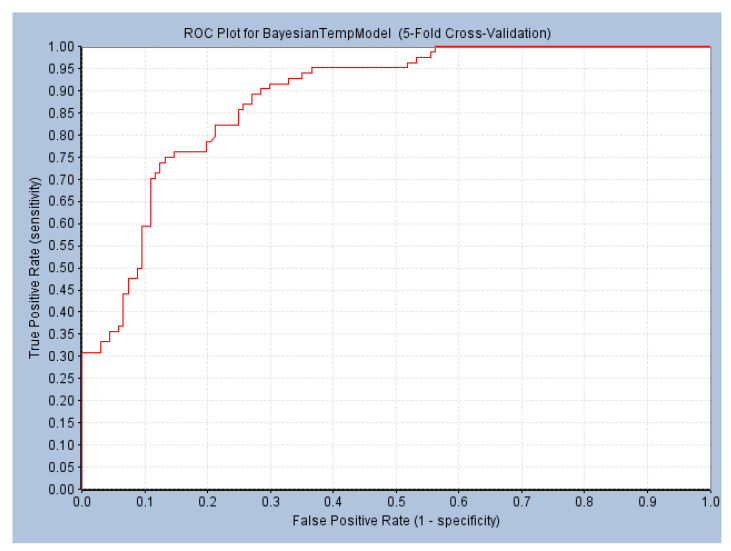
ROC curve obtained by five-fold verification of the NB model. (ROC = 0.884; AUC = 0.878).

**Figure 3:**
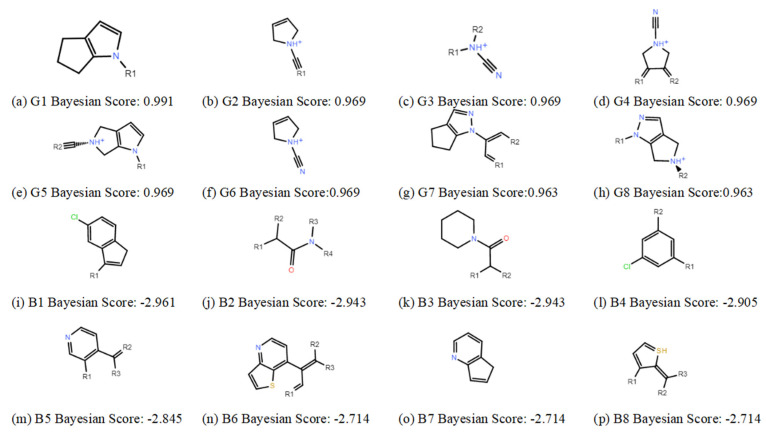
Favorable/unfavorable active fragments calculated based on ECFP_6: (**a**–**h**) top 8 good fingerprints (G1–G8) calculated based on ECFP_6 fingerprints; (**i**–**p**) top 8 bad fingerprints (B1–BF8) calculated based on ECFP_6 fingerprints.

The Naive Bayes (NB) model shows excellent classification recognition ability. For the classification of active molecules, the true positive rate (recall rate) reached 75.6%; for inactive molecules, the model has a prediction rate of 84.0% for true positives (inactive molecules classified correctly). The AUC of this model is 0.878, close to 1, indicating that this model has good classification ability. The classifier model is slightly better at identifying inactive molecules than active ones. This result shows that classification using this model can increase the risk of incorrectly excluding active compounds. However, more false positives can be avoided. Because we used more screening and validation methods throughout the screening process, the results were acceptable. The results obtained in the five validation tests of the model are similar to the classification results after training. As can be seen from Figure 2, in our 5-fold cross-validation results, the model also shows a good ROC-AUC index. On the basis of the ECFP_6 fingerprint, high-frequency good/bad feature fragments (GF/BF) of active and inactive compounds were calculated. Since the molecular fingerprint frequencies of all compounds classified as “active” by Bayesian classifiers are taken into account when calculating favorable fragments, the fragments in Figure 3 represent only the statistical results of a large data-based study of the structure-activity relationship of potential USP7 inhibitors, contributing to our understanding of the key structure of USP7 inhibitors.

#### 2.1.3. Construction and Verification of Multiple Linear Regression Model

**Figure 4:**
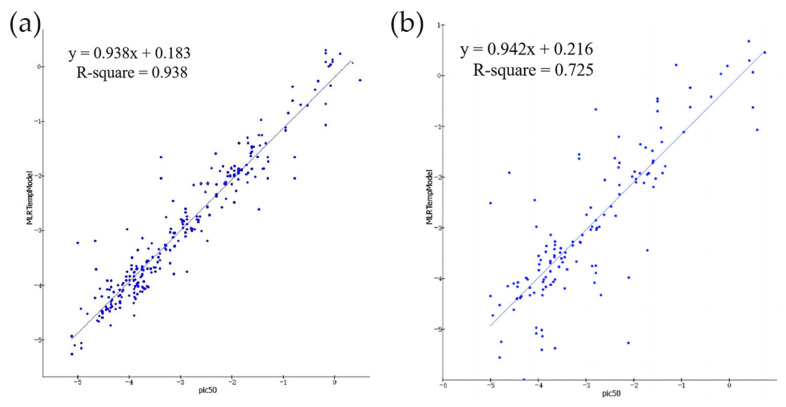
Correlation between predicted and true values of the MLR model. (**a**) The correlation between the predicted and true values of the training set. (y = 0.938x + 0.183, R^2^ = 0.938); (**b**) Correlation between the predicted and true values of the test set. (y = 0.942x + 0.216, R^2^ = 0.725).

As shown in Figure 4, the MLR model constructed by DS has good performance. In the real and predicted values of compounds, R2 of the training set is 0.938 and R2 of the test set is 0.725. Therefore, we chose this model for the pIC50 classification of Marine compounds. We screened a total of 232 effective small molecules. As with the AutoQSAR model, we chose small molecules with pIC50 > 6. There are 10 of them in total, and they are included in the 240 clocks screened in the AutoQSAR model. They will undergo docking analysis of Maestro, GOLD, and MOE in turn.

### 2.2. The Analysis of Molecular Docking 

The good performance of a molecular docking tool is essential for structure-based screening and analysis of the interaction forces between protein–ligands. In order to treat the molecular docking process with more rigor, the binding site of its eutectic ligand was re-docked with extra precision (XP) using the USP7 protein (PDBID: 6M1K) prior to true covalent docking. When the docked small molecule and the primary crystal ligand are superimposed, the RMSD values between them are calculated. If the RMSD value is less than 2.0Å, the molecular docking tool is considered to perform well. The primary eutectic ligand and the re-docking ligand show that the small molecules after the re-docking are very close to the primary eutectic ligand. The RMSD between Maestro, GOLD, and MOE were 0.3234Å, 0.778Å, and 0.5480Å, respectively. Thus, this proves that Maestro, GOLD, and MOE are trusted docking tools.

In order to find suitable covalent inhibitors of USP7, we performed XP docking before covalent docking. This is because covalent docking consumes more computer power and time than XP docking. At the same time, this can also successfully enter the active pocket of the chemical compounds screened out. Also, in Scaffold hopping, it is very important to consider the flexibility of the receptor. If not considered, it can hinder the discovery of correct posture in docking [31]. It is therefore reasonable to perform XP docking before covalent docking. The results showed that 240 small molecules could be successfully docked, and 78 compounds were better than the positive control. In addition, a second XP docking after Scaffold hopping was also successfully performed. Therefore, the 78 compounds and the optimized compounds will undergo the next covalent docking.

According to GOLD docking results, positive control P217564 (reference compound) produced a total of 20 conformations, and its average CHEMPLP value was 35.0956, which was lower than that of the other 15 small molecules, so covalent docking was performed on these 15 small molecules.

MOE docking results showed that P217564 (the reference compound) and the optimized compound were successfully docked. However, only optimized compound 1008-1 showed results due to positive control compounds, and optimized compound 1008-1 was also the only docking analysis that could pass three different docking procedures simultaneously. Therefore, optimized compound 1008-1 has great potential to become a covalent inhibitor of USP7.

### 2.3. Covalent Docking

**Figure 5:**
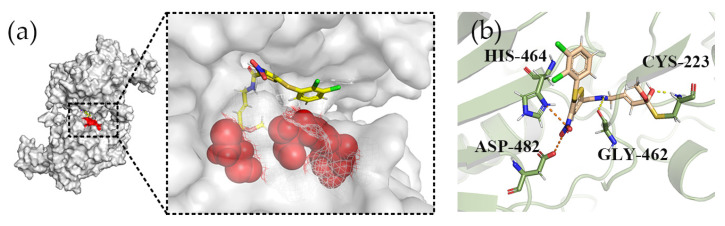
The analysis of the positive compound P217564 (reference compound) and protein pocket. (**a**) The relationship between the positive control compound P217564 (reference compound) and the surface of the active pocket (the red part is the location of the active pocket); (**b**) the three-dimensional binding pattern of P217564 (reference compound) to USP7 protein (PDBID: 6M1K). The hydrogen bond interaction is yellow. The salt bridge is orange. The covalent interaction is directly linked to the residue (CYS223).

The pharmacological advantages of covalent inhibitors are being studied extensively. Covalent inhibitors can not only obtain a longer drug residence time but also improve the selectivity of the target. In the USP7 protein structure, the core consists of amino acids CYS223, HIS464 and ASP481, which together participate in the process of substrate deubiquitination, so it is also known as the catalytic domain. As shown in Figure 5a, the catalytic domain of USP7 is a typical hand-like structure, where the positive control is bound in the gap between the thumb and the palm, and this region is also a key region that guides the C-terminal of ubiquitin to bind to the active site. The covalent docking fraction between P217564 (the reference compound) and protein was −5.583kcal/mol. As shown in Figure 5b, compound P217564 (reference compound) forms hydrogen bond interactions with residues CYS223 and GLY462, and salt bridge interactions with ASP482 and HIS464. Therefore, compound P217564 (the reference compound) can be used as a reliable control compound.

In XP docking, 78 compounds were selected for covalent docking with the USP7 protein. The docking fraction and docking pattern of 22 marine compounds were better than those of the positive control compound P217564 (reference compound), so these compounds were used for scaffold hopping, thereby improving the interaction between ligand and protein. The optimized structure of thousands of compounds is once again XP docking and covalent docking. All compounds target the cysteine residue CYS223 for covalent binding. According to different compounds, different covalent reaction equations are chosen. Finally, we found that 58 compounds with optimized structures had better docking scores and docking patterns than positive controls and original small molecules. Therefore, they were selected for further ADMET property analysis to find more stable USP7 covalent inhibitors.

According to the covalent docking results of GOLD, the positive control P217564 (reference compound) formed three conformations, and its average CHEMPLP value was −70.12986667, and the average CHEMPLP value of six small molecules was lower than that of P217564 (reference compound).

### 2.4. Scaffold Hopping

The main purpose of scaffold hopping is to enhance the physicochemical and pharmacological properties of the original drug. Scaffold hopping is mainly based on the binding effect of the pocket and the molecule. The diversity of fragment binding resulted in a large number of newly generated molecules, and the replacement results of each molecule showed a large amount of data—more than 100. Therefore, we restrict according to the requirements of the pharmacophore of the original molecule, molecular weight, and lipid solubility to meet better pharmacological properties. The GBVI/WSA dG score was used to evaluate the binding effect between the structurally optimized small molecule and the original pocket. We performed scaffold hopping on each of the 22 molecules. Through conditional restriction, score screening, and visual observation, 22 groups of small molecules with optimized structures were obtained for XP docking and covalent docking again. As shown in Table 2, better GBVI/WSA dG scores were obtained after scaffold hopping through fragment replacement. In particular, compound 13057 had the same structure “1,2-dibromobenzene” as the positive control compound, and it was observed that this structure did not interact well with the key residue during the covalent docking visualization. However, according to the binding relationship between MOE molecules and pockets, the exposed area represented by 13057-3 is smaller than 13057-1 and 13057-2. However, due to the consideration of the substitution principle and the rigor of the experiment, we carried out different selection schemes of pharmacophore for the two parts, respectively. In order to find compounds with better structure, the structurally optimized compounds were compared before and after a second XP docking and covalent docking.

### 2.5. Analysis before and after Scaffold Hopping

**Figure 6:**
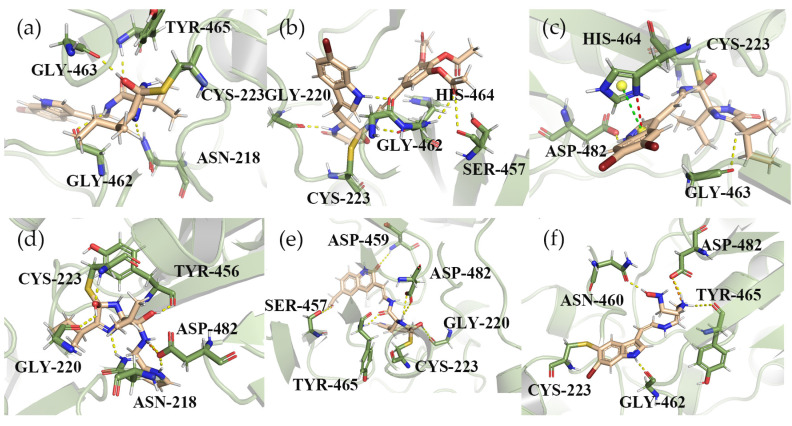
Three-dimensional binding patterns between protein-ligand complexes. (**a**) Three-dimensional structure of the protein complex of compound 13058. (**b**) Three-dimensional structure of compound 1008 and protein complex. (**c**) Three-dimensional structure of the protein complex of compound 13057. (**d**) Three-dimensional structure of the optimized compound 13058-2 protein complex. (**e**) Three-dimensional structure of the structurally optimized compound 1008-1 and protein complex. (**f**) Three-dimensional structure of the optimized compound 13057-3 protein complex. (Hydrogen bond interactions are yellow, cation-π interactions are red, π-π interactions are green, salt-bridge interactions are orange, and covalent interactions are directly connected to residues CYS223.).

The three compounds with good covalent docking results are shown in Figure 6 and Table 3, which respectively show the three-dimensional binding patterns of complexes before and after the skeleton transition and the interactions of key residues. All the compounds can covalently interact with CYS223, a key residue of the catalytic domain. In particular, the structurally optimized compound 13058-2 interacts with residue CYS223 and hydrogen bonds, which makes it better bound to the protein. Both compound 1008 and the optimized compound 1008-1 have the same interaction with GLY220, SER457, and CYS223. Although the covalent docking scores of compounds 1008 and 1008-1 are close, compound 1008-1 is superior to compound 1008 in the docking modes of their respective complexes. Both compound 13057 and compound 13057-3 form hydrogen bond interactions with ASP482 residues, but in particular, compound 13057-3 also forms salt bridge interactions with ASP482, forming a stronger binding force. Therefore, the optimized compounds 13058-2, 1008-1, and 13027-3 are better than the original compounds and the positive control compound.

**Figure 7:**
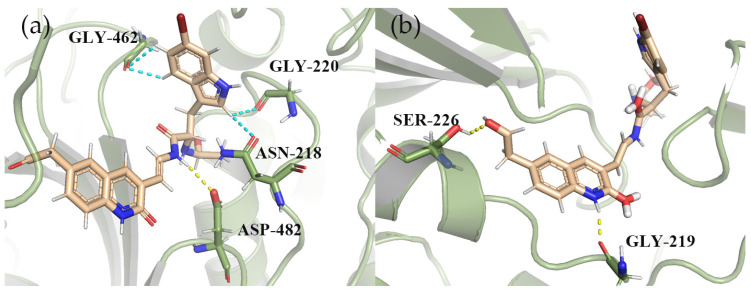
The three-dimensional binding mode between compound 1008-1 and protein after optimization. (**a**) three-dimensional combination mode using MOE docking; (**b**) 3D bonding mode using GOLD docking. (The hydrogen bond interaction is yellow, and the Aromatic H bond interaction is cyan).

To further confirm the reliability of our selected structures, we used GOLD and MOE software for docking, and the optimized structure of 1008-1 was able to obtain better results than the positive compound P217564 (reference compound) in three different docking software. As shown in Figure 7a, the docking results of 1008-1 in MOE software showed that the compound formed the Aromatic H bond interaction with residues GLY462, GLY220, and ASN218, and the hydrogen bond interaction with residues ASP482. The interactions of the residues above are basically consistent with the docking results shown with Maestro. As shown in Figure 7b, the compound interacts with residues SER226 and GLY219 to form hydrogen bonds. After comprehensive analysis of the docking results of Maestro, MOE, and GOLD, the optimized compound 1008-1 has potential candidate compound capability.

### 2.6. Property Analysis of ADMET

The online website ADMETlab2.0 was used to predict the absorption, distribution, metabolism, excretion, and toxicity of 58 compounds after structural optimization. Some physical and chemical properties of positive control compound P217564 (reference compound) exceeded the threshold, such as LogP exceeding the upper limit and LogS exceeding the lower limit. The other compounds selected after structural optimization are within the appropriate range of physical and chemical properties. As shown in Table 4, the ADMET properties of the structurally optimized compounds were compared with the positive control compounds. Madin-Darby canine kidney cells (MDCK) have been developed as an in vitro model for permeability screening. Compounds 1008-1, 13057-3, 13058-2, and P217564 (the reference compound) all showed good MDCK values, indicating that they enter the body more efficiently. Compounds 1008-1, 13057-3, and P217564 (the reference compound) have a lower blood-brain barrier. Volume distribution (VD), which relates the administered dose to the actual initial concentration present in circulation, is an important parameter to describe the distribution of a drug in the body. Compounds 1008-1, 13057-3, 13058-2, and P217564 (reference compound) all showed good VD values. In addition, liver damage from drugs is a common side effect of drugs. The H-HT value of compound P217564 (reference compound) is 0.547, which is higher than that of compounds 1008-1, 13057-3, and 13058-2. Therefore, the three optimized compounds 1008-1, 13057-3, and 13058-2 have good properties for patent medicine.

### 2.7. The Analysis of RMSD and RMSF

**Figure 8:**
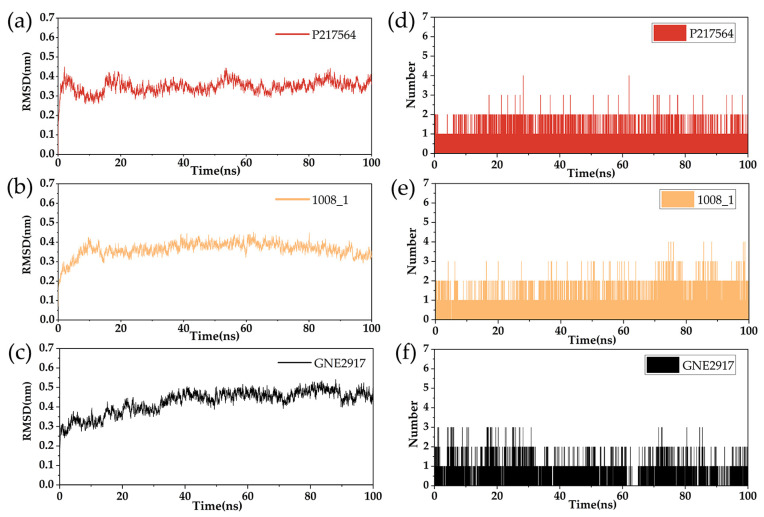
RMSD and hydrogen bond analysis diagram of the complexes of USP7 and compounds. (**a**) RMSD of USP7 and positive control compound P217564 (reference compound). (**b**) RMSD of USP7 and compound 1008-1. (**c**) RMSD of USP7 and compound GNE2917 (negative control compound). (**d**) Hydrogen bond number of USP7 and compound P217564 (reference compound). (**e**) Hydrogen bond number of USP7 and 1008-1. (**f**) Hydrogen bond number of USP7 and GNE2917 (negative control compound).

The RMSD value reflects the degree to which the atoms deviate from the average position, that is, the size of the motion of each atom. The RMSD of the protein-ligand complexes is shown in Figure 8. The RMSD value of compound P217564 (the reference compound) fluctuated slightly between 18ns and 55ns, but remained stable overall. The RMSD of compound 1008-1 was stable after 15ns. The RMSD value of GNE2917 (the negative control compound) tended to stabilize after 40ns, but the RMSD value after its equilibrium was higher than that of P217564 (the reference compound) and 1008-1. The average RMSD of the complexes of protein and P217564 (reference compound), 1008-1, and GNE2917 (negative control compound) were 0.3447, 0.3637, and 0.4081, respectively. Compared with P217564 (the reference compound), 1008-1 has a similar RMSD value, while the RMSD value of GNE2917 (the negative control compound) was higher than that of P217564 (the reference compound) and 1008-1.

For candidate compounds 1008-1, the fluctuation of RMSD after reaching stability is not more than 2 nm, which indicates that the complex can finally reach a relatively stable state during the simulation process.

**Figure 9:**
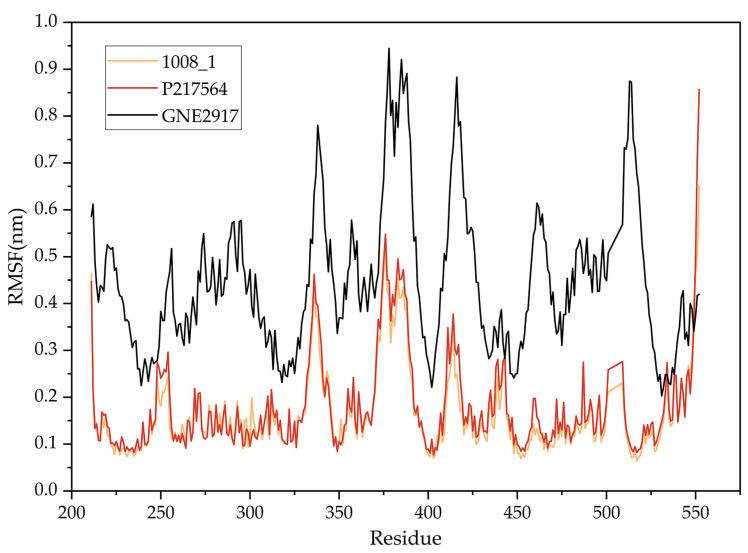
RMSF diagram of USP7 with compounds.

The RMSF calculates the fluctuations of the individual amino acid residues during MD simulation with respect to time, which reflects the degree of freedom of the atom. The RMSF of the protein is shown in Figure 9. In general, the overall movement trend of the P217564 (reference compound) is consistent with that of 1008-1, while the GNE2917 (negative control compound) has a higher RMSF value. RMSF shows lower values near residues ASP482, CYS223, and TYR465, which indicates that the protein has a more stable structure near these residues. This is consistent with the results of molecular docking.

### 2.8. Hydrogen Bond Analysis

The hydrogen bond between the protein and the ligand is an important factor in keeping the molecule within the active site cavity. During the simulation process, the ligand forms a certain number of hydrogen bonds with the protein, and the number of these hydrogen bonds also reflects the degree of binding of the ligand to the protein. As shown in Figure 8, the number of hydrogen bonds formed by compound 1008-1 within 100 ns is stable. Compared with 1008-1, GNE2917 (the negative control compound) had fewer hydrogen bonds with the protein, indicating that 1008-1 has better hydrogen bond interactions with the protein. The results of RMSD, RMSF, and hydrogen bond analysis indicate that 1008-1 has good binding stability.

## 3. Discussion

USP7 is a human ubiquitin-specific protease 7, also known as the herpes virus-associated protein. It has been shown to regulate the stability of different cellular proteins, which play an important role in DNA replication and transcription, apoptosis, and the immune response [32]. USP7 is the target of the herpes virus protein that enables the virus to escape and replicate efficiently, playing multiple roles in the process of viral infection [33]. At the same time, it is a key factor in the p53 pathway. In the p53-MDM2-USP7 pathway, USP7 maintains a sufficient MDM2 level, resulting in a low level of p53, thus creating conditions for the occurrence and development of tumors [34]. As a result, USP7 has become a pandemic target for many diseases. Recognizing the importance of USP7 in the p53 pathway, it was reported in 2006 that USP7 plays an important role in the carcinogenesis of non-small cell lung cancer (NSCLCs) through the p53-dependent pathway [35]. USP7 is highly expressed in a variety of cancers and affects cancer development. This has fueled research interest in USP7 inhibitors [36]. Based on experimental and molecular docking methods, USP7 inhibitors of the natural primary crystal triterpene skeleton were reported in 2018 and demonstrated inhibitory effects on myeloma cell proliferation [37]. Molecular dynamics simulations and biological evaluation techniques were used to determine that USP7 inhibitors had an inhibitory effect on LNCaP in human prostate cancer cells. Despite the fact that many USP7 inhibitors have been reported, there are currently no drugs available for purchase. Therefore, small molecules that can inhibit USP7 for clinical treatment have yet to be discovered. Fortunately, computer technology has advanced to the point where CADD can be used to screen potential small molecules from large compound libraries, allowing for a more efficient and cost-effective drug screening process with a higher hit rate of potential compounds.

Covalent inhibitors are a class of compounds that can covalently bind to specific target proteins, thereby inhibiting their biological functions. Targeted covalent inhibitors (TCI) have continued to innovate in recent years and have expanded beyond cysteine-directed electrophiles, kinases, and cancers, offering broad opportunities for a new generation of breakthrough therapies [38]. In 2020, the structurally active study of electrophilic peptide inhibitors in the catalytic domain of USP7 was reported, and it was found that inhibitors with 1-Cyanopyrrolidine warheads can promote the β-elimination reaction of covalent admixtures [39]. Based on the highly conserved catalytic triad structure of USP7 protein, cysteine residue CYS223 was covalently added with different chemical shells to induce conformation changes at the active site, thereby inhibiting its enzyme activity [5]. Reported FT827 contains a vinyl sulfonamide structure extending towards the catalytic center and covalently modifies USP7 cysteine. Therefore, it is reasonable to design covalent compounds of cysteine targeting USP7 to provide novel inhibitors of USP7 derived from marine compounds.

Quantifying the binding affinity between small molecules and their targets is essential for modern drug discovery. To predict the activity of the Marine compound library, we collected small molecules with high or low activity of USP7 inhibitors from BindingDB and built three different QSAR models: the AutoQSAR model, Naive Bayesian model, and the MLR model. The best model, kpls_radial_20, had the smallest difference between R^2^ and Q^2^, indicating its extensive and robust nature. In Naive Bayesian model, we found that pyrrole structure can play a positive role in active action. Three kinds of molecular docking procedures were performed based on Marine compounds co-screened by three different QSAR models. We also evaluated the performance of the docking software and conducted scaffold hopping to optimize the covalent docking results over positive control compounds to enhance the binding ability of the compounds to proteins and their stability in dynamic simulations. The three optimized compounds, 13058-2, 1008-1, and 13057-3, were covalently bonded. Compounds 1008-1 and 13058-2 underwent a Nucleophilic Addition to a Double Bond, while 13057-3 underwent a Nucleophilic Substitution. The replaced fragment of the compounds enhanced the interaction with the protein. Although 13057-1 and 13057-2 had the same 1,2-dibromobenzene structure as the positive control compounds and achieved better scores on the covalent butt and GBVI/WSA dG scores, they did not dominate the ADMET property analysis and molecular dynamics simulation. Thus, changing the 1, 2-Dibromobenzene structure may improve the binding effect. At the same time, the optimized compound 1008-1 showed good interaction with residues ASP482, GLY220, and GLY220 in different docking procedures. The screened compounds formed more hydrogen bonds with USP7 than positive compounds, and the key binding residues were similar, suggesting that optimized compound 1008-1 may be a covalent inhibitor of USP7. Molecular dynamics simulations of the complex showed the dynamics of ligand–protein binding and confirmed the results of static covalent docking. Through a comprehensive analysis, we have concluded that these three compounds possess the desired qualities of target binding and pharmaceutical properties, thereby making them ideal candidates for the further development of USP7 covalent inhibitors.

## 4. Materials and Methods

### 4.1. Compound Data Set Preparation

To be able to collect data on reported USP7 inhibitors with experimental activity, we collected data on 667 compounds with IC_50_ activity values from the publicly accessible database BindingDB. (https://www.bindingdb.org/rwd/bind/index.jsp, accessed on 6 February 2023) In order to ensure the integrity of the compound data and to process the subsequent steps of the data, the small molecules with incomplete data information were deleted, resulting in 543 compounds. In addition, the second-generation USP7 inhibitor P217564 (reference compound) [40] was selected as a positive control compound in this study due to its high potency and selectivity, its ability to selectively target the catalytic crack of USP7, and its ability to modify cysteine at the active site by forming a covalent adduct. For all compounds, the Maestro 11.8 (Schrodinger, Shanghai, China) Structure File Converter is used for file format conversion of small molecules, and the Glide module is used for small molecule preparation. In this step, all the small-molecule structures were desalted at pH 7.0 ± 2.0 using the OPLS_2005 force field and Epik module using LigPrep tools to generate 3D structures with corresponding low-energy states and their isomers.

### 4.2. Protein Crystal Structure Preparation

Human ubiquitin-specific protease 7(PDB ID: 6M1K) was downloaded from the PDB database (https://www.rcsb.org/, accessed on 19 September 2022). In the Protein Preparation Wizard tool in Maestro 11.8, the protein crystal structure is prepared. In order to make the protein crystal structure more refined, the PH value was set to 7.0, and the protonation state of the protein residues was optimized by hydrogen bonding using PROKAs pKa prediction. In addition, in order to better simulate the effect, the OPLS_2005 force field was used to minimize the energy of the protein structure and limit the heavy atoms of the protein to the RMSD value of 0.3 Å. We will use the prepared small-molecule structure and protein structure for further USP7 inhibitor screening.

### 4.3. The Construction of Three Different QSAR Models

#### 4.3.1. Construction and Prediction of AutoQSAR Model

Maestro 11.8’s AutoQSAR tool is a powerful automated application that builds QSAR models based on machine learning methods and has been used to build QSAR models that combine affinity with reliable performance comparable to published QSAR models. [41] In the construction of the AutoQSAR model, 543 small molecule structures prepared by LigPrep are input as the learning set of the model, and the inhibitory activity of all small molecules [IC_50_(nmol/L)] is transformed into a negative logarithmic scale [pIC50(mol/L)]. Small molecules with different IC_50_ values across different orders of magnitude were randomly assigned 75% as the training set and 25% as the test set. It then computes 497 physicochemical and topological descriptors as well as various canvas fingerprints, producing a large pool of independent variables. But because descriptors often contain a high degree of redundancy, a feature selection process is performed to identify a smaller subset of descriptors. If these descriptors of the learning set do not provide statistically significant information when at least 90% of the compounds in the learning set have the same value, then these descriptors are removed. After initial filtering, the set of descriptors still exhibits significant collinearity, and some machine learning methods can effectively reduce redundancy, such as recursive segmentation and partial least squares regression. The filtered descriptors are further reduced to no descriptor pairs exhibiting absolute Pearson correlation coefficients. A subset of 0.8. The number of models to be constructed for each model type is set to 50. Multiple regression algorithms integrating optimal subset multiple linear regression (MLR), partial least squares regression (PLS), kernel-based least squares regression (KPLS), and principal component regression (PCR) are used to construct numerical models. And according to S.D. (Standard deviation of the model), R^2^ (coefficient of determination for the training set), RMSE (root mean-square error of the test set predictions), and Q^2^ (the R-squared for the test set), statistical parameters and composite scores were used to select the best numerical models for AutoQSAR. The best-evaluated AutoQSAR model was used to screen marine natural compounds for potential USP7 inhibitors.

#### 4.3.2. Construction and Prediction of Naive Bayesian Model

The discrimination ability of machine learning classification models depends heavily on whether the chemical spatial distribution of the compounds in the training data set is sufficiently diverse. Therefore, the chemical spatial diversity of 55 calculated descriptors from the entire data set was investigated using a principal component analysis (PCA) analysis. The PCA of the molecular descriptors yielded eight key molecular descriptors, namely ES_Count_aasN, ES_Count_aaO, ES_Count_dCH2, Num_AromaticRings, Molecular_FractionalPolarSurfaceArea, IsChiral, Num_Rings, and QED. Five principal components (PCs) were obtained by assigning distinct weighting factors to these descriptors. The first three most critical principal components (PC1–PC3) were selected for the chemical spatial analysis. Bayesian classification models were constructed using 737 active/inactive usp7-targeted compounds. The key molecular descriptors obtained from the dimensionality reduction in the PCA, as well as the molecular fingerprint ECFP_n, were applied to the proposed model construction process. Various fingerprint-descriptor combinations were attempted during the model construction, whereas the area under the ROC curve (AUC) values of the obtained models were considered as indicators representing their classification ability. The best-performing Bayesian model was selected for further analysis, in which an active flag was used for classification. The naive Bayesian model is based on a binary (i.e., ‘yes or no’) approach to determining whether a compound has target inhibitory activity, with the classification process using the model’s activity cut-off value (−4.000 in our model) as a benchmark, along with a Bayesian score for each compound. When the absolute value of the Bayesian score is greater than the cut-off value, the compound is classified as ‘active’, and the opposite is classified as “inactive”. Subsequently, ECFP_6 and eight key descriptors were applied (ES_Count_aasN, ES_Count_aaO, Num_AromaticRings, Molecular_FractionalPolarSurfaceArea, IsChiral, Num_Rings, ES_Count_dCH2, and QED).

#### 4.3.3. Construction and Prediction of Multiple Linear Regression Model

Through extensive literature review and the use of databases, we collected 481 molecules structure and bioactivity data related to 2D QSAR. Dividing them into training sets and test sets. We use computational chemistry to calculate 2D molecular descriptors for each molecule, including the topological structure, charge distribution, and solubility. These descriptors are used as the basis for constructing quantitative structure-activity relationships. The pic50 value was used as the activity data. Using the Create Multiple Linear Regression Model in Discovery Studio, we construct 2D QSAR models using advanced modeling techniques. The MLR QSAR model was used to predict the biological activity of 240 molecules.

### 4.4. Structure-Based Virtual Screening

#### 4.4.1. Molecular Docking Using Maestro

In order to further screen the USP7 covalent inhibitors, super-precision docking (XP) and covalent docking (covalent docking) were performed on the candidate compounds based on the USP7 structure (PDB ID: 6M1K). For molecular Docking studies using the Maestro 11.8 software, the pre-prepared USP7 protein crystal structure was placed into the Ligand Docking tool for XP docking. However, before starting the docking with the Glide module, the scoring function and docking parameters are verified in advance. In order to make the docking result more accurate, the docking mode is set to flexible docking. Here, to soften the potential of the non-polar part of the ligand, set the scale factor to 0.80 and the partial charge cutoff value to 0.15. Other than that, to soften the potential of the non-polar portion of the protein structure, the van der Waals radius scaling factor and partial charge cutoff values of the receptor are set to 1.0 and 0.25, respectively. Therefore, 240 marine compounds and scaffold hopping compounds screened from the AutoQSAR model were coupled with the USP7 protein structure with additional precision in order to better determine the protein-ligand binding site and bring the ligand close to the covalent residue CYS223.

To screen for covalent inhibitors of the USP7 catalytic domain, we restricted the ligand to within 10Å of the catalytic triad of the receptor (CYS223, HIP464, ASP481). On the basis of the structure after XP docking, the virtual screening of optimized marine compounds was carried out. In addition to analyzing the ranking of ligand-receptor covalent docking scores, all complex binding patterns were visually examined. Compared with the positive control compound P217564 (reference compound), better compounds were screened for further analysis and optimization.

#### 4.4.2. Molecular Docking Using MOE

Molecular Operating Environment (MOE) docking software (MOE2019.0102) was used for molecular docking. The compounds screened by the first two Maestro and GOLD were moleculetically docked with the positive compound P217564 (reference compound). We use MOE to minimize the energy of a three-dimensional geometry for small molecules. The protein structure was optimized with A root-mean-square gradient of 0.1kcal/mol/A and the QuickPrep minimization constraint retained. We used Triangle Matcher as the placement method. We use the London dG scoring function to rank the docked conformations because the function estimates the binding free energy of the ligand for a given conformation. And we calculate the docking method by calculating the root-mean-square deviation (RMSD) between the primary ligand and the redocking ligand. Three kinds of docking consensus judgments were used to determine the candidate compounds with good potential.

#### 4.4.3. Molecular Docking Using GOLD

GOLD 5.3 (Genetic Optimization for Ligand docking 5.3) was used for docking. USP7 and a novel inhibitor complex (PDBID:6M1K) were used as receptors to add hydrogen atoms and remove water molecules. CYS223, HIS464, and ASP481 were set as binding sites, and chemscore_kinase was selected as an available Template. The GA Runs of P217564 (the reference compound) were set to 20. The GA Runs of the other 15 molecules are set to 10, and CHEMPLP is selected as the docking score. In order to improve the calculation accuracy, the Search efficiency was set to Very Flexible, and molecular docking was started.

In addition, GOLD was used for covalent docking with CYS223 as the reaction residue, and chemscore_kinase was selected as an available Template. The GA Runs of positive control P217564 (reference compound) were set to 20, the GA Runs of the other 15 molecules were set to 10, and CHEMPLP was selected as the docking score. Ligand link mode Select the Atom option to define the Protein link atom and the Ligand link atom. Similarly, to improve the accuracy of the calculation, we set the Search efficiency to Very Flexible.

### 4.5. Scaffold Hopping by Fragment Replacement

Scaffold hopping is a common strategy for drug structure optimization. Changes in the position and number of heteroatoms in the molecular skeleton based on fragment design can greatly affect the physicochemical properties, bioactivity, and pharmacokinetics of small molecules. Therefore, the rational use of scaffold hopping strategies based on target structure can improve the success rate of drug candidates [42,43].

#### 4.5.1. Molecular Processing

We chose the calculation software MOE, and the fragment database we selected was the fragment library that came with MOE (40,626 fragments). In order to achieve a better binding effect between protein and ligand, partial replacement of the molecular skeleton was performed according to artificial judgment to achieve an optimal effect. First, we looked at the 2D representation of the binding effect of the molecule to the protein pocket presented by MOE. Secondly, based on the ligand interaction identified by MOE, we analyzed the exposed regions of the molecule and the locations of the special bonds between the molecule and the pocket. Finally, the area with a large exposure area and less binding force is selected manually. When the box selection area is wrapped in green, the selected area is recognized as replaceable by MOE. In addition, in order to ensure that the molecules after the transition are not less effective than the original molecules, pharmacophore restriction should be applied to the selected hydrogen bonds or large pie bonds to preserve them if necessary. For most of the transition molecules, we give the following uniform restriction conditions: Weigh < 500, SlogP [–4, 8], TPSA [40, 140], thereby limiting the newly formed molecule, and when the molecular weight of the selected structure increases significantly, we change the molecular weight to 600 to achieve better results.

#### 4.5.2. Optimization and Scoring

In order to better judge the binding effect of the original pockets, GBVI/WSA dG is used as a scoring function to score the conformation of the newly generated molecules and order the resulting molecules (scaffold hopping part) [44]. The higher the absolute value of the calculated scores, the better the binding effect of the small molecules with the original pockets. After the computer gives the ranking, we uniformly select the small molecules whose score is above 10 in absolute value. When the overall score is high or low, we select the top 100 or so molecules for subsequent calculation.

### 4.6. Prediction of ADMET Properties

It can be known from past experience that predicting the ADMET (Absorption, Distribution, Metabolism, and Toxicology) properties of compounds is an extremely important process for drug discovery. In this study, the ADMETlab2.0 online website (https://admetmesh.scbdd.com/service/screening/index, accessed on 23 April 2023) was used to measure the ADMET properties of compounds [45]. The site can import SMILES files or SDF files to predict drug properties, including physicochemical properties, pharmacochemical properties, and pharmacokinetics. Based on the predicted results, a better compound than the positive control compound P217564 (reference compound) was selected for further Dynamics simulation analysis.

### 4.7. Molecular Dynamics Simulations

In order to further test the stability of the candidate compounds in the binding pocket, molecular dynamics simulations were performed using the GROMACS2019.1 software package. We used the Bio2byte Web server to generate topology files for the candidate compounds and positive control compounds (https://www.bio2byte.be/acpype/, accessed on 8 October 2023) [46,47]. The AMBER99SB-ILDN force field and the SPC216 water model were used for molecular dynamics simulation [48,49]. To ensure the electrical neutrality of the reaction system, a corresponding number of sodium ions were added to the system to replace water molecules. Periodic boundary conditions were applied to the three directions of the spatial coordinates of the complex system. Set the analog temperature to 300 k, and the energy minimization of the system is carried out in 50,000 steps. After correcting the position constraints, the isothermal volume (NVT) and isothermal pressure (NPT) were adjusted, respectively, to achieve the equilibrium of the acceptor, ligand, and solvent. We selected 1008-1, which has better ADMET properties and a better docking score than the positive control compound P217564, for MD simulation with a duration of 100ns, while using the structurally similar compound GNE2917 [4] as the negative control. Each simulation process was repeated three times to ensure the reliability of the data.

## 5. Conclusions

USP7 is closely related to the occurrence and development of many cancers and is a promising anticancer target. In this study, three different QSAR models were first constructed: the optimal AutoQSAR model kpls_radial_20 and the MLR model were constructed by the regression method, and the Naive Bayesian model was constructed by the classification method. The QSAR model was used for preliminary screening of Marine compound libraries. Next, after the screening of the AutoQSAR model and the MLR model, compounds with pIC50 greater than 6 were selected for molecular docking. At the same time, the positive control compound P217564 (reference compound) was compared, and the better compound was selected for Scaffold hopping. The molecular docking was then performed again, comparing it not only with P217564 (the reference compound) but also with the compound before the structural optimization. Then, 58 compounds with optimized structures were selected to predict ADMET properties. We selected 10 compounds for kinetic simulation and took the average three times to observe the stability between the compounds and proteins. Based on the above analysis results of marine compounds and compounds with optimized structures, compound 1008-1 has great potential to become a novel covalent inhibitor of USP7, providing a new possibility for accurate treatment of USP7-related diseases.

## Figures and Tables

**Table 1:** Statistical results of the numerical AutoQSAR models of the top 10.

Model Code	Score	S.D.	R^2^	RMSE	Q^2^
kpls_radial_20	0.7847	0.5998	0.8077	0.6184	0.8016
kpls_radial_5	0.7827	0.5797	0.8224	0.6101	0.8018
kpls_linear_20	0.7776	0.4559	0.8889	0.5662	0.8337
kpls_dendritic_20	0.7765	0.4491	0.8922	0.5648	0.8345
kpls_dendritic_34	0.7700	0.5324	0.8501	0.6018	0.8048
kpls_linear_5	0.7641	0.5264	0.8528	0.6050	0.8051
kpls_linear_34	0.7613	0.5377	0.8471	0.6116	0.7984
kpls_radial_13	0.7598	0.5320	0.8498	0.6110	0.8050
kpls_molprint2D_13	0.7501	0.5263	0.8545	0.6161	0.8017
kpls_dendritic_5	0.7500	0.5276	0.8522	0.6178	0.7967

S.D.: Standard deviation of the model; R^2^: R-squared value (coefficient of determination) for the training set; RMSE: Root-mean-square error of the test set predictions; Q^2^: Q-squared value (the R-squared for the test set).

**Table 2:** The results of scaffold hopping through fragment replacement. (The replacement structure is marked in pink).

Name	Pharmacophore Limitation	Filter Criteria	Before Scaffold Replacement	After Scaffold Replacement	Score
1008-1	No	Weight < 600, SlogP [–4, 8], TPSA [40, 140], Score less than −12	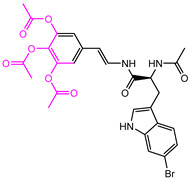	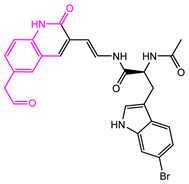	−18.4846
24428-35	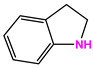	Weight<500, SlogP [–4, 8], TPSA [40, 140]	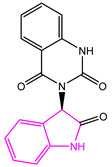	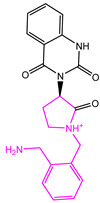	−13.1977
13058-2	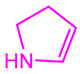	Weight < 500, SlogP [–4, 8], TPSA [40, 140], Score less than −10	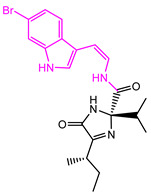	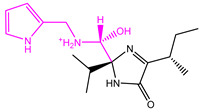	−10.7885
13058-3				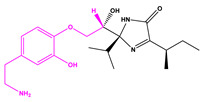	−13.0436
13057-1	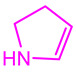	Weight < 500, SlogP [–4, 8], TPSA [40, 140]	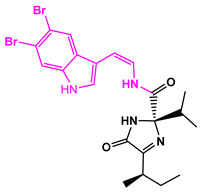	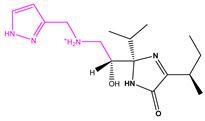	−11.2398
13057-2				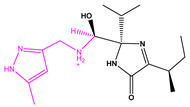	−11.4333
13057-3	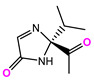		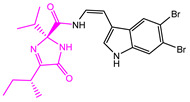	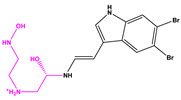	−8.5368
8171-3	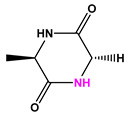	Weight < 500, SlogP [–4, 8], TPSA [40, 140]	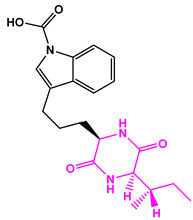	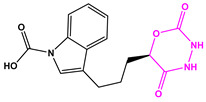	−14.2391
8171-6				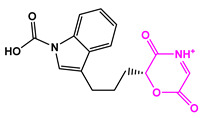	−12.9634
8171-7				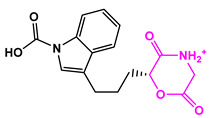	−12.4163

**Table 3:** The interaction of compounds with key residues of proteins.

Compound	Hydrogen Bonds	π-π	Cation-π	Salt Bridge	Covalent Docking Score (kcal/mol)
P217564	CYS223, GLY462	-	-	ASP482, HIS464	−5.583
13058	TYR465, GLY463, GLY462, ASN218	-	-	-	−5.866
13058-2	CYS223, TYR465, GLY220, ASN218, ASP482	-	-	ASP482	−7.865
1008	GLY220, GLY462, GLY483, SER457, HIS464	-	-	-	−7.852
1008-1	ASP459, ASP482, GLY220, TYR465, SER457	-	-	-	−7.868
13057	ASP482, GLY463	HIS464	HIS464	-	−5.820
13057-3	ASN460, ASP482, TYR465, GLY462	-	-	ASP482	−6.549

**Table 4:** ADMET results for compound P217564 and the scaffold-optimized compounds.

Compound	MDCK	BBB	VD	Fu	CYP2C19-Sub	H-HT	AMES	EC	EI
1008-1	8.34 × 10^−6^	0.071	1.813	29.79%	0.053	0.116	0.442	0.003	0.006
13057-3	2.48 × 10^−5^	0.063	0.954	65.57%	0.065	0.134	0.007	0.006	0.012
13058-2	3.48 × 10^−6^	0.355	1.011	67.65%	0.064	0.062	0.007	0.003	0.019
P217564	3.15 × 10^−5^	0.016	1.196	1.65%	0.423	0.547	0.947	0.003	0.043

MDCK Permeability: high permeability > 20 × 10^−6^ cm/s, medium permeability for 2–20 × 10^−6^ cm/s, low permeability for < 2 × 10^−6^ cm/s; BBB: blood brain barrier, 0–0.3 cm/s: excellent, 0.3–0.7cm/s: medium, 0.7–1.0 cm/s: poor; VD: Volume Distribution, 0.04–20 L/kg: excellent; otherwise: poor; Fu: The fraction unbound in plasms >20%: High Fu, 5–20%: medium Fu <5%: Low Fu; CYP2C19-sub: the probability of being substrate/inhibitor, within the range of 0 to 1; H-HT: The human hepatotoxicity, 0–0.3: excellent, 0.3–0.7: medium, 0.7–1.0: poor; AMES: The Ames test for mutagenicity, 0–0.3: excellent, 0.3–0.7: medium, 0.7–1.0: poor; EI/EC: The eye irritation/corrosion, 0–0.3: excellent, 0.3–0.7: medium, 0.7–1.0: poor.

## Data Availability

The data used to support the findings of this study are included within the article.
